# Atypical Teratoid/Rhabdoid Tumor with Retained *SMARCB1* (*INI1*) Expression and Rare *SMARCA4* Gene Mutation: A Case Report of a Pediatric Patient

**DOI:** 10.3390/reports7020028

**Published:** 2024-04-22

**Authors:** Anna Marija Mališkina, Ivanda Franckeviča, Zelma Višņevska-Preciniece, Marika Grūtupa, Žanna Kovaļova

**Affiliations:** 1 Faculty of Medicine, Riga Stradins University, Dzirciema Street 16, LV-1007 Riga, Latvia; 2 Department of Pathology, Riga Stradins University, 9c Kuldīgas Street, LV-1007 Riga, Latvia; 3 Department of Pathology, Children’s Clinical University Hospital, LV-1004 Riga, Latvia; 4 Department of Hematology and Oncology, Children’s Clinical University Hospital, LV-1004 Riga, Latvia; 5 Department of Pediatrics, Children’s Clinical University Hospital, LV-1004 Riga, Latvia

**Keywords:** atypical teratoid/rhabdoid tumor, retained *INI1*, *SMARCA4*

## Abstract

Atypical teratoid/rhabdoid tumors (AT/RT) are highly aggressive tumors of the central nervous system (CNS), accounting for 1–3% of all pediatric CNS tumors. In general, AT/RTs are associated with biallelic inactivation of *SMARCB1*, resulting in the loss of expression of the integrase interactor 1 (*INI1*) protein. In this report, we describe the clinical course of an infant patient who presented with fatigue, postprandial vomiting, and disability of left side movement. Histological examination revealed classical features indicative of rhabdoid tumors, yet an atypical immunohistochemical profile with preserved *INI1* expression was observed. Molecular diagnostics further elucidated the presence of a heterozygous frameshift variant, *SMARCA4* c.2693del, p.(Asn898Thrfs*12), underscoring the distinctive genetic foundations of the case. Surgical resection of the tumor was administered with subsequent chemotherapy to the patient, but the condition worsened dynamically, and a decision was made to give the patient palliative therapy. We report on a patient with AT/RT caused by a rare mutation of the *SMARCA4* gene and an aggressive course of disease to provide more information and characteristics of these tumors.

## 1. Introduction

Atypical teratoid/rhabdoid tumor (AT/RT) is a highly aggressive central nervous system (CNS) malignancy, found mainly in pediatric patients. Worldwide, AT/RT constitutes 1–3% of all pediatric central nervous system tumors and represents 20% of CNS tumors in children under 3 years of age [1,2,3].

Ordinarily, AT/RT is associated with biallelic inactivation of the *SMARCB1* (SWI/SNF-related matrix-associated actin-dependent regulator of the chromatin subfamily B member 1) gene at position 22q11.2. [4,5]. AT/RT can be broken down into three different molecular groups: AT/RT-SHH, AT/RT-TYR, and AT/RT-MYC. In general, these groups differ from each other in age at the time of diagnosis, location in the brain, epigenetic profile, and prognosis [1,2,3,5,6]. The *SMARCB1* gene encodes the integrase interactor 1 *INI1* protein, being a core member of the adenosine triphosphate-dependent SWI/SNF (SWItch/sucrose non-fermentable) chromatin remodeling complex, which possesses a wide-ranging function in activating genes by remodeling nucleosomes and facilitating the accessibility of transcription factors to their respective recognition sites, thus acting as a key regulator of cell proliferation and cell lineage determination [3,7]. In rare cases, mutations are observed in the *SMARCA4* gene, which encodes another member of the SWI/SNF chromatin remodeling complex—named Brahma-related gene 1 (*BRG1*)—that facilitates transcriptional activation or repression of target genes and acts as a tumor suppressor through chromatin remodeling [8]. In these cases, *INI1* expression is typically preserved and does not rule out a diagnosis of AT/RT; thus, it is necessary to determine *BRG1* nuclear expression [1,4].

The diagnosis of AT/RT presents challenges that are attributable to the absence of distinctive symptoms and radiological features, the variability in its anatomical location within the brain, and the histopathological and cytogenetic complexities associated with this condition. To determine the diagnosis, it is necessary to perform an immunohistochemical examination for the *INI1* or *BRG1* proteins, as well as a genetic examination for AT/RT-associated genes [9,10,11,12].

There is a standardized protocol available for treating AT/RT, established by the European Rhabdoid Registry (EU-RHAB). The ongoing assessment involves a multimodal therapeutic approach encompassing surgery, systemic and intraventricular chemotherapy, and radiation therapy [13,14].

In this report, we describe the aggressive clinical course of an infant patient with retained *INI1* expression and absent nuclear labeling for *BRG1*, in order to provide further insight into the clinical, immunohistochemical, and genetic characteristics of these tumors.

## 2. Detailed Case Description

A 10-month-old baby was admitted to the emergency room after her parents expressed concern about her inability to lean or move her left arm. An X-ray of the clavicle and the proximal part of the upper arm was performed, where no fractures were found, and the child was discharged from the outpatient clinic. Two days later, the patient returned to the emergency department with vomiting and liquid stools. The patient’s blood and urine tests did not reveal significant deviations from the norm; the patient received symptomatic therapy and was subsequently discharged. However, the patient exhibited progressive nausea and postprandial vomiting, accompanied by pronounced lethargy, diurnal somnolence, and notable deficits in motor functions, including an inability to lift the legs bilaterally and inadequate grip strength, which was particularly evident on the left side. After two similar episodes, the patient was hospitalized for an in-depth investigation. At the time of hospitalization, the general condition of the patient was fair, with stable vital signs and a Glasgow Coma Scare score of 15, but paresis of the left side of the body was observed. According to the neurologist’s instructions, magnetic resonance imaging (MRI) was performed, and a large tumor was found. The description was as follows: a large heterogeneous pathological mass lesion in the dorsobasal part of the right frontotemporal lobe with medial growth, various age hemorrhages with wide hemorrhagic cystic cavities in the upper part of the formation in the frontal lobe with pronounced midline dislocation, ventricular compression, and periventricular edema. In the initial stage, computed tomography of the chest cavity and ultrasonography examination of the abdominal cavity did not show the presence of metastases.

**Figure 1:**
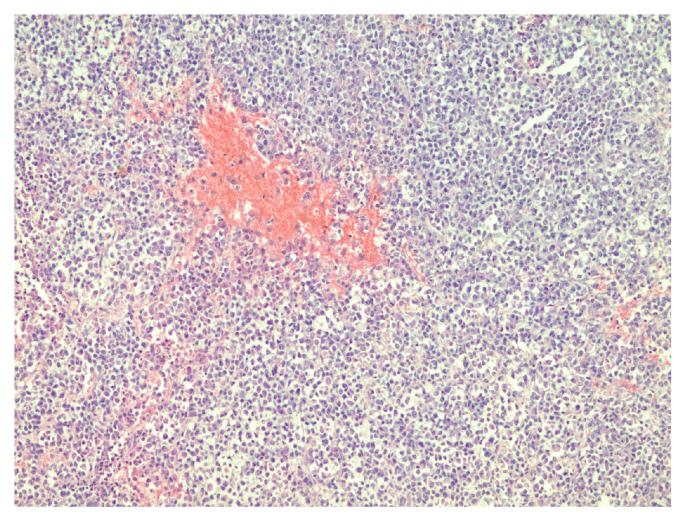
Densely packed tumor cells and focal hemorrhages in tumor tissue, hematoxylin–eosin, original magnification ×100.

**Figure 2:**
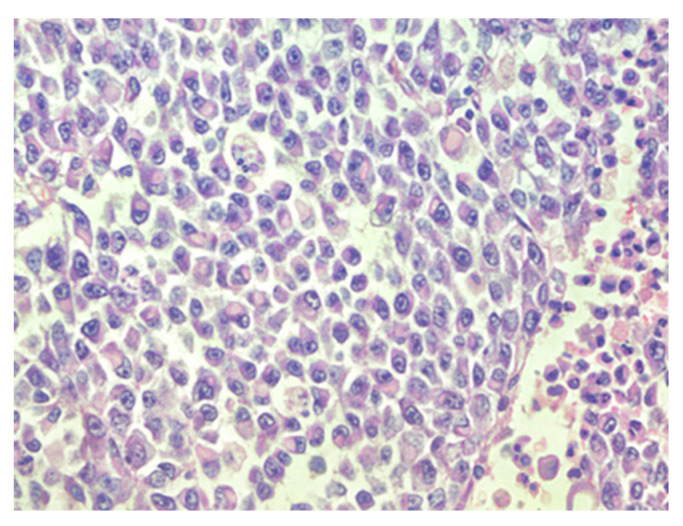
Sheets of rhabdoid cells with eccentrically located nuclei and eosinophilic cytoplasm, hematoxylin–eosin, original magnification ×400.

**Figure 3:**
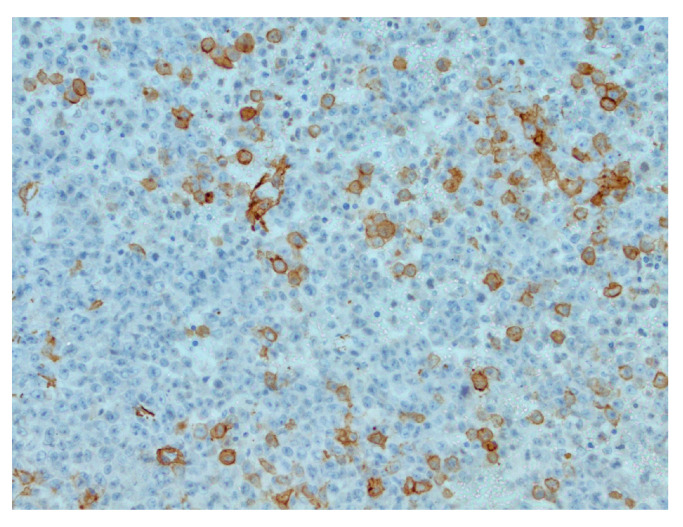
Focal actin positivity in tumor cells, IHC (Immunohistochemistry), original magnification ×200.

**Figure 4:**
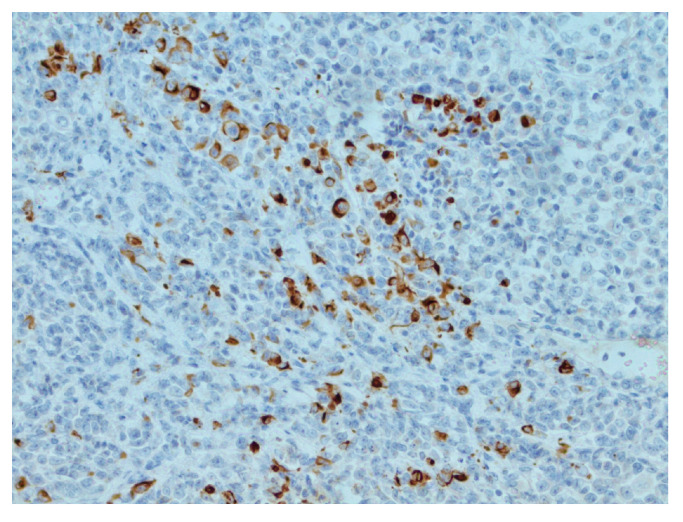
Focal CKAE1/AE3 positivity in tumor cells, IHC, original magnification ×200.

**Figure 5:**
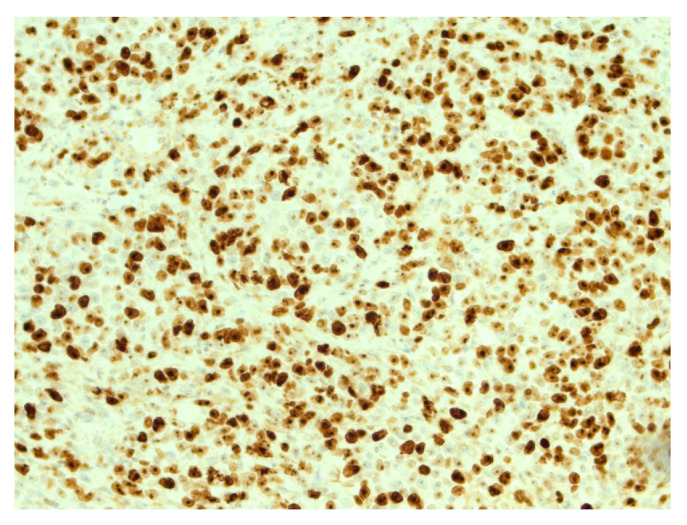
High expression of Ki67 in tumor cells. IHC, original magnification ×200.

**Figure 6:**
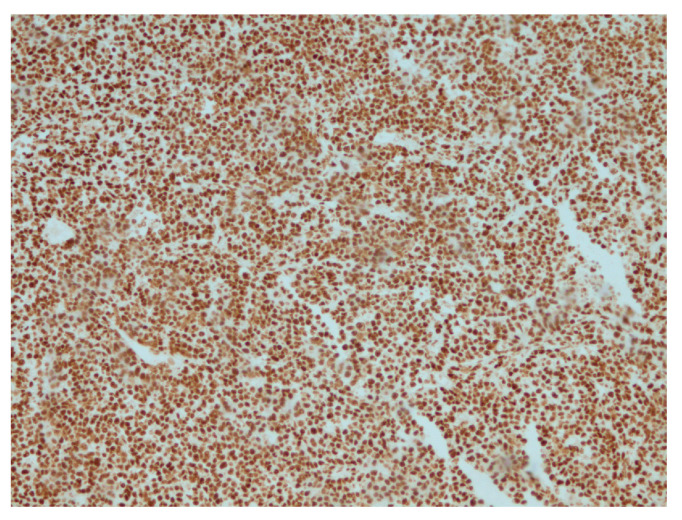
Retained expression of INI-1 (*SMARCB1*) in tumor cells, IHC, ×200.

The patient was transferred to a neurosurgical facility, where a subsequent right-sided craniotomy with subtotal resection was performed. Total resection was not feasible as the tumor infiltrated the M1 segment of the right middle cerebral artery (MCA). Subsequently, the patient’s condition showed a dynamic improvement, and she started to move her left arm and leg more. The histological picture was consistent with the histology of the classic rhabdoid tumor: a small, round-shaped cell with an eccentrically placed nuclear, dense chromatin pattern, and bright eosinophilic cytoplasm. Immunohistochemical analysis showed retained nuclear expression of *INI1*, which is not a typical AT/RT finding. The proliferative marker Ki67 reached 90% at a higher concentration of positive cells, suggesting aggressive progression of tumor growth. The antibodies used for immunohistochemistry were sourced from respected suppliers and underwent extensive validation procedures to ensure reliability and specificity. Specifically, INI1 and BRG1 antibodies were obtained from Santa Cruz Biotechnology Company, while antibodies targeting EMA (epithelial membrane antigen), SYN (synaptophysin), GFAP (glial fibrillary acidic protein), S-100 and Ki67 were sourced from Dako Corporation. Additionally, the IDH1 (isocitrate dehydrogenase 1) antibody was obtained from Dianova. The pathological and immunohistochemical analysis of the patient’s surgical specimen is shown in Figure 1, Figure 2, Figure 3, Figure 4, Figure 5 and Figure 6.

DNA methylation of CpG islands is a key feature in various tumors [15]. An Illumina 850k EPIC Array analysis was performed at the National Hospital for Neurology and Neurosurgery (NHNN) in London to obtain the characteristic pattern of DNA methylation. Tumor DNA was extracted and modified in-house, and the generated data were uploaded to the brain tumor methylation classifier 11b4. The obtained methylation class family was atypical teratoid/rhabdoid tumor, subclass SHH. O(6)-methylguanine-DNA methyltransferase (MGMT) promoter status was unmethylated. Additional immunostaining for *BRG1* was performed, which was not available at the initial immunohistochemical examination stage. As a result, the absence of nuclear labeling of *BRG1* in tumor cells was detected.

Furthermore, the patient’s blood sample was examined using next-generation sequencing analysis at a certified clinical laboratory. Sequence reads from each sample were mapped to the human reference genome (GRCh37/hg19). Mutation calling was performed using The Genome Analysis Toolkit (GATK)algorithms (Sentieon) for nDNA. The Blueprint Genetics (BpG) Hereditary Pediatric Cancer Panel identified a heterozygous frameshift variant, *SMARCA4* c.2693del, p.(Asn898Thrfs*12), which has not been previously described in the medical literature, reported in disease-related variation databases such as ClinVar or HGMD, and is absent in gnomAD. The acquired data proved that the genetic changes were associated with rhabdoid tumor predisposition syndrome. The reporting process was carried out using HUGO Gene Nomenclature Committee (HGNC)-approved gene nomenclature and mutation nomenclature following the Human Genome Variation Society (HGVS) guidelines.

The family was counselled to undertake genetic analysis encompassing the parents and siblings. Upon conducting a genetic examination of the parents, a pathogenic variant in the *SMARCA4* gene was identified in the patient’s mother’s sample. The family was briefed on the options and implications of in vitro fertilization, along with the need for genetic counseling during 8 to 11 pregnancy weeks in the event of a natural pregnancy.

A month after surgery, intravenous and intraventricular chemotherapy were prescribed, and a Porth-a-Cath was surgically implanted in the patient, as well as an Ommaya reservoir for drug delivery. Chemotherapy was provided to the patient according to the European Rhabdoid Registry (EU-RHAB) protocol. Regrettably, subsequent magnetic resonance assessments following chemotherapy and surgical intervention indicated evidence of prolonged tumor growth. There was also periventricular confluent demyelination of the corpus callosum, along with demyelination of the splenium, that may have been associated with therapy. The patient experienced significant difficulties tolerating all chemotherapy treatments and encountered severe side effects, including prominent pancytopenia, dyspeptic complaints, severe mucositis, febrile neutropenia, and sepsis. In the last course of chemotherapy, doses of ifosfamide and etoposide were reduced. The clinicopathological characteristics are summarized in Table 1.

Through consultation with the Nordic Association of Pediatric Hematology and Oncology (NOPHO), it was decided to provide palliative therapy. Symptomatic palliative care was provided to the patient. Unfortunately, the patient passed away nine months after the diagnosis was determined.

The pathological and immunohistochemical analysis of the patient’s surgical specimen (Figure 1, Figure 2, Figure 3, Figure 4, Figure 5 and Figure 6).

## 3. Discussion

AT/RT may present with equivocal symptoms, making accurate interpretation challenging. In the case described, the patient exhibited movement deficiencies at an early age, later accompanied by progressive vomiting. Several differential diagnoses could present with these symptoms. Our case highlights the challenges in diagnosing and interpreting clinical presentations. This underscores the necessity for neurological examination in similar cases, which is crucial for excluding less common but severe pathologies and ensuring prompt treatment.

The council recommended symptomatic palliative therapy, considering several negative factors. The presented patient was 10 months old, and the data from the literature reveal that the reported age at the time of diagnosis has previously been established as a crucial prognostic determinant [16,17,18]. The subsequent unfavorable prognostic factor was the impossibility of total resection due to vascular infiltration with tumor tissue. Lafay-Cousin et al., in their study, have shown that there is a statistically significant difference in outcomes between subtotal and complete total resection [19]. Heterozygous germline pathogenic variants in the *SMARCB1* and *SMARCA4* genes have been reported to cause rhabdoid tumor predisposition syndrome (RTPS), which is characterized by a high risk of malignant rhabdoid formation with the most frequent localizations in the central nervous system or kidneys and is linked to a more unfavorable prognosis compared to those with sporadic rhabdoid tumors [20,21]. In the presented case, a germline mutation was also found in the patient’s blood sample. As a result of the above-mentioned facts, several aggressive treatment risks outweighed any potential benefits against the short, expected survival. Firstly, the significant size and localization require a large volume of radiation therapy. Furthermore, the risk of bleeding is further complicated by the presence of hemorrhages in the tumor nodes. Further risks were associated with therapy, including side effects on the developing nervous system and other organ systems. The parents understood the recommendations, confirmed that they did not want the child’s quality of life to be reduced if it was not possible to cure the disease, and agreed to cooperate with the Palliative Care Service.

Unfortunately, there is no way to protect the child from developing a tumor if genetic changes have occurred in the *SMARCA4* gene. Consequently, there are only secondary prevention options associated with aggressive treatment and its side effects [21]. Routine examinations are imperative for the prompt identification of metastases and the onset of novel neoplasms in diverse organs, a hallmark feature of cases associated with RTPS. Genetic counseling is necessary for all families that have had cases with such a pathology, as it will allow them to decide the need for prenatal testing and pre-implantation options.

Holdhof et al. have published a case series of 14 AT/RT tumors hosting *SMARCA4* mutations, describing their distinct characteristics from *SMARCB1*-deficient cases [5]. Compared to *SMARCB1* alteration, AT/RT cases caused by the pathogenic variant of the *SMARCA4* gene have been linked to a higher prevalence of germline mutations, younger age, and a worse prognosis [5,22]. Our reported case provides additional data reflecting an aggressive course with a poor prognosis in a patient with a *SMARCA4* mutation. The patient was 10 months old at the time of diagnosis. During discussions with parents, it was noted that the patient may have experienced left-sided movement disturbances earlier in life. However, these limitations did not significantly impact the patient’s daily activities and thus did not warrant special attention. This suggests that the tumor probably began developing several months earlier and is relevant to the literature data that *SMARCA4* gene-associated cases occur at a young age. The implications stemming from this factor lead to a worse prognosis. The youthful age constrains the potential application of radiotherapy due to its high neurotoxicity. Nevertheless, reviews in the available literature indicate that radiation therapy can significantly affect patient survival [23,24,25,26]. In the new The European Society for Pediatric Oncology (SIOPE) ATRT01 randomized study, which was not in place at the time of the patient’s treatment, the minimum age for radiation therapy is reduced to 12 months [26].

Our presented case reflects the aggressive prognosis of an AT/RT tumor against the background of all therapy and emphasizes that *SMARCA4* mutation-causing cases do not have a favorable prognosis.

According to the 2021 World Health Organization Classification of CNS Tumors, atypical teratoid/rhabdoid tumors are classified in the category of ‘other central nervous system embryonal tumors’, being Grade 4 tumors [27]. Confirming the presence of an atypical teratoid rhabdoid tumor by pathology is crucial, as the MRI results and clinical manifestation often resemble those of other high-grade brain tumors in young patients [10]. It is important to recognize the differential diagnosis, such as high-grade glial tumor, anaplastic ependymoma, choroid plexus carcinoma, and medulloblastoma [1,22,28]. Consequently, confirmation of certain specific genetic variations is necessary for a diagnosis. The distinctive histological features, coupled with immunohistochemical evidence demonstrating the loss of nuclear expression of *INI1*, frequently provide adequate confirmation for diagnosing AT/RT. However, *INI1* loss can also manifest in other cancers, and a small proportion of AT/RTs may still express *INI1*, which generally complicates the determination of the diagnosis [1,10,11]. Our clinical case presentation underscores the significant relevance of molecular diagnosis, particularly in scenarios where *INI1* remains immunohistochemically intact despite the histological manifestation that resembles a rhabdoid tumor. In the study conducted by Holdhof et al., only half of the analyzed *SMARCA4* AT/RT cases were clearly identified as AT/RT subgroups through methylation analysis [5]. Our findings provide supplementary evidence underscoring the utility of methylation analysis in practical clinical settings for the diagnosis of AT/RT. DNA methylation analysis is indispensable not only for elucidating the diagnosis in cases where the immunohistochemical profile is atypical but also for furnishing supplementary insights into the molecular subtype of the tumor. This information is instrumental in advancing research related to the treatment modalities and prognosis associated with these tumors.

## 4. Conclusions

AT/RT is a rare tumor; however, it is more common in pediatric patients than in adults and is characterized by a poor prognosis and outcome. Given that atypical teratoid/rhabdoid tumors induced by the *SMARCA4* gene mutation are a rare and aggressive type of cancer, they are associated with an unfavorable prognosis, and the exact prevalence is difficult to define. Some studies have mentioned that pathogenic variants of the *SMARCA4* gene mutations cause AT/RT in up to 2% of cases [10,29]. We present a case report of the aggressive course of AT/RT with a molecularly identified heterozygous frameshift variant *SMARCA4* c.2693del, p.(Asn898Thrfs*12), which, to our knowledge, has not been previously described in medical literature. Our presented clinical case demonstrates the importance of testing additional elements of the SWI/SNF chromatin remodeling complex. Furthermore, the case also underscores the role of molecular diagnostics, incorporating methylation analysis and the identification of germline mutations. Additionally, it highlights the significance of genetic testing within familial cohorts to rule out rhabdoid tumor predisposition syndrome; thus, emphasizing the crucial need for genetic testing in both diagnosing and predicting outcomes.

## Figures and Tables

**Table 1:** Summary of the general characteristics.

General Characteristics		
Age at Diagnosis	10 months	
Gender	Female	
Clinical Manifestation	Postprandial vomiting Nausea Neurological deficiency	
Tumor location	Supratentorial	
Immunohistochemical staining	INI1	Positive
	EMA	Negative
	SYN	Positive
	GFAP	Negative
	S-100	Negative
	BRG1	Negative
	IDH 1	Negative
SMARCA4 pathogenic variant	c.2693del, p.(Asn898Thrfs*12)	
Methylation class	AT/RT-SHH	
Survival	9 months	

## Data Availability

The data presented in this study are available on request from the corresponding author due to protection of patient privacy.
